# Supplementary material for: Field- and laboratory-based studies on correlates of Chlamydia trachomatis transmission by Musca sorbens: Determinants of fly-eye contact and investigations into fly carriage of elementary bodies
Source: PLoS Negl Trop Dis. 2024 Jul 2;18(7):e0012280. doi: 10.1371/journal.pntd.0012280 (PMC11249242; doi:10.1371/journal.pntd.0012280)
Supplement: S1 Table — (DOCX) [file pntd.0012280.s001.docx]

# Supplementary information

The two forms of the *Musca sorbens* species complex that occur in Africa can be differentiated by the width of the frons. In *M. sorbens*, the males have a relatively broad frons (0.079-0.118 X the maximum head width), while in *M. biseta*, the males have a relatively narrow frons (0.042-0.076 X the maximum head width). The frons to head width ratio found in males of our *M. sorbens* colony ranges from 0.08-0.11 (Table 1).

Table 1. Head and frons measurements of ten males from our M. sorbens colony at the London School of Hygiene & Tropical Medicine.

| Head number | Head width | Frons width | head width mm | frons mm | ratio |
| --- | --- | --- | --- | --- | --- |
| 1 | 80 | 8 | 0.80 | 0.08 | 0.10 |
| 2 | 79 | 8 | 0.79 | 0.08 | 0.10 |
| 3 | 75 | 8 | 0.75 | 0.08 | 0.11 |
| 4 | 78 | 8 | 0.78 | 0.08 | 0.10 |
| 5 | 85 | 9 | 0.85 | 0.09 | 0.11 |
| 6 | 85 | 8 | 0.85 | 0.08 | 0.09 |
| 7 | 75 | 8 | 0.75 | 0.08 | 0.11 |
| 8 | 85 | 8 | 0.85 | 0.08 | 0.09 |
| 9 | 75 | 6 | 0.75 | 0.06 | 0.08 |
| 10 | 78 | 8 | 0.78 | 0.08 | 0.10 |
