# Supplementary material for: Field- and laboratory-based studies on correlates of Chlamydia trachomatis transmission by Musca sorbens: Determinants of fly-eye contact and investigations into fly carriage of elementary bodies
Source: PLoS Negl Trop Dis. 2024 Jul 2;18(7):e0012280. doi: 10.1371/journal.pntd.0012280 (PMC11249242; doi:10.1371/journal.pntd.0012280)
Supplement: Attachment — Submitted filename: Trachoma Review.pdf [file pntd.0012280.s002.pdf]

Dear Editorial staff / Authors,

I have reviewed the manuscript PNTD-D-24-00098, entitled "Further studies on the transmission of Chlamydia trachomatis by Musca sorbens in Oromia, Ethiopia"; The manuscript seems well organized, but kindly you can find some points needs to be revised by the respectable authors, as below:

1- The type of study should be mentioned in the "methods" part.
2- The manuscript needs to be improved for English language.
3- In abstract it has been written "*Fly-eye contact was positively associated with the presence of trachoma disease, lower body weight and increased body temperature*." But the body weight loss and fever have not been defined in this survey.
4- As mentioned in line 10: "*the role of Chlamydia trachomatis as vectors has been convincingly demonstrated via randomised controlled trials in the Gambia"*, why this study has been performed on a well-known fact?
5- In line 37 "The authors found evidence of on Ct on flies and…" the proposition "on" has been repeated twice.
6- Considering the Oromia region of Ethiopia as the place of research performing, and contribution of some respected authors outside Ethiopia, it seems better that the role of each author be specified in the manuscript.

Best regards,
Reviewer
