# Supplementary material for: Field- and laboratory-based studies on correlates of Chlamydia trachomatis transmission by Musca sorbens: Determinants of fly-eye contact and investigations into fly carriage of elementary bodies
Source: PLoS Negl Trop Dis. 2024 Jul 2;18(7):e0012280. doi: 10.1371/journal.pntd.0012280 (PMC11249242; doi:10.1371/journal.pntd.0012280)
Supplement: Attachment — Submitted filename: Manuscript PNTD-D-24-00098 Reviews .doc [file pntd.0012280.s003.doc]

Manuscript PNTD-D-24-00098 Reviews

Further studies on the transmission of Chlamydia trachomatis by Musca sorbens in Oromia, Ethiopia

The manuscript PNTD-D-24-00098 by Robinson et al presents a combined field and laboratory study evidence of *Musca sorbens* as potential vectors of Trachoma in Oromia region of Ethiopia, whose knowledge was not quite well understood. These findings are relevant to the disease field, and in particular, to guide designs of controlling the disease transmission. This data is a follow-up from the same research group meant to advance the disease transmission mechanisms by the flies, whereby the proximity to infected hosts was described to influence the flies abundance and associated with transmission rates of the trachoma disease, further delineated by laboratory infections and a scanning microscopy.

In my keen assessment, whilst the manuscript is well written and data analyzed, the manuscript requires substantial revisions before acceptance.

Major concerns:

The authors failed to deduce the main points from the data that communicate to the scientific community and the title to my understanding requires to capture that. Change the title to suit the obtained data.

Was there a negative control region for the flies study design?

With regards to the study design of the lab experimental, the data lacks surety of whether the acquired bacterium could be transmissible for no evidence to a suitable animal model, and the authors failed to acknowledge this or cite it as a limitation. Further, if proximity of infection hosts was key to transmission rates, this was not proven or attempted or just ignored in the experimental design. Additionally, the proposed tests in lines 493 – 497 are doable to strengthen manuscript, and therefore recommended.

Minor comments

**Abstract**

Line 8 ….. are thought to be vectors of

Line 14 delete ‘flies’ in the brackets

I**ntroduction**

Line 71: What is the meaning of ‘E’ interventions, not defined

**Methods**

Study location and design: How many times were the surveys conducted and does this impact how the data was analyzed? What kind of sampling strategy was adopted and the rationale considered? How close or far from each other were the households chosen, and why? These questions were not clearly answered.

Line 136: How were the flies caught killed? Simply say acetone killing

Line 156: Clearly define the meant specific ‘testing’

The authors need to clearly state the number of independent replicates conducted for the fly experiments. Why was the laboratory experiments conducted with very few flies? Does this not limit the power of the

Fly samples for Ct testing: The authors wrote that ‘knocked-down flies were first washed and transferred into bleach….’ This experiment does not mimic the natural transmission phenomenon. For if the external bacterium on the flies’ surface was washed off, then not detectable for mechanical transmission to be noticed experimentally, or not independently assayed (then contradicts the results lines 377 – 382). Why bleach the samples from the lab and fail to do the same for those from the field, yet a comparison is expected to be made? Any valid reasons?.

Statistical analysis

(1) Authors failed to inform how all the fly catches irrespective of infection status were analyzed, based on locations for fly density before stratifying for infection positivity, which is stated in lines 268 – 276. I recommend re-analysis.
(2) What was the importance of collecting video data of fly-contacts yet the flies could be counted manually, and or was not presented.

**Results**

As earlier on stated, the data is sufficiently analyzed, well presented, and described.

Line 306 …… higher on individuals

Line 352 - 367 A total of 107 (and not 106) flies (refer to line 281 that stated 24/107). Make the necessary corrections
