# Supplementary material for: Field- and laboratory-based studies on correlates of Chlamydia trachomatis transmission by Musca sorbens: Determinants of fly-eye contact and investigations into fly carriage of elementary bodies
Source: PLoS Negl Trop Dis. 2024 Jul 2;18(7):e0012280. doi: 10.1371/journal.pntd.0012280 (PMC11249242; doi:10.1371/journal.pntd.0012280)
Supplement: Attachment — Submitted filename: Response_to_reviews_Robinson_2024.docx [file pntd.0012280.s004.docx]

Thanks very much to all three reviewers, your comments have helped strengthen the manuscript so we very much appreciate the time you put into reading the paper so carefully. Please find our point by point responses below

**Reviewer 1**

- Lines 39-40 : "that M. sorbens flies contribute to transmission of the disease”. Strictly speaking, they contribute to transmission of the infection.
  - Thank you for highlighting this error, we have amended
- 42: please change “regions” to “populations” – “regions” has a political element, and the prevalence is found in a human population
  - Thank you for this useful suggestion, we have made this change
- 47: “is the leading cause of infectious blindness worldwide” – please change to “is the leading infectious cause of blindness worldwide”: the blindness itself is not infectious
  - Thank you for this useful suggestion, we have done this
- 55. “the species ratio is always predominantly”, suggest change to “the species ratio of flies found at the eye is always predominantly”
  - Thank you for this useful suggestion, we have made this change
- 139-140 and 148-149: the authors note that Virkon-S sterilized the nets and dissecting equipment, but does that solution degrade the DNA sequences of interest?
  - Virkon-S has been shown to be effective in removing DNA,^1^ this has been noted in the text (L166)
- 150: Five flies per child were… Should this read, “Up to five flies per child were…”?
  - Yes, this is strictly speaking more correct – there were so many flies caught that there were very few instances of fewer than five!
  - Amended, L176
- 160: this seems to contradict 148-149
  - These are two different experiments, using two different methods; one (*Fly identification and testing for Ct DNA (whole flies)*) was done at the time of the survey immediately after fly capture. The second (*Fly testing for Ct DNA (body parts)*) was done retrospectively at a different location, in our molecular testing laboratory. Both methods of sterilisation achieve the same results.
- 196: Does the parenthetical text indicate that ice was used to kill the flies?
  - L222 – clarified
- 247 and 257: The words “The effect of…” implies causality, whereas I think all that’s being looked for here is an association
  - Thanks for spotting this, we have changed the wording from “effect of” to “association between” in both instances, as well as a third instance where we were looking at the association between household distance and percentage of Ct-positive flies caught. (L267)
- 345: suggest change “ear (body)” to “body (ear)”
  - Thank you, see L378
- 348: the word “in” is missing I think
  - Thank you, see L381
- 419-420: “This is indicative that weight is important to fly contacts, with the lightest children experiencing the most contacts”. This suggests volition on the part of the flies.
  - The phenomenon of insects being preferentially attracted to some individuals within a population is well documented across many species and does not suggest volition but is a natural outcome of their olfactory-based host seeking preferences. Insects locate their hosts using olfaction (and additionally vision in diurnal species). When certain hosts produce more of the volatile chemicals that insects are attracted to, those individuals are naturally more attractive to the host-seeking insect. This happens for many reasons, for example changes in body temperature, different body mass, innate differences in skin chemistry and, sometimes, the presence of disease. In many vector borne disease systems there is increasing evidence that the infectious disease agent either changes/manipulates the skin odour to enhance attraction, or exploits a fortuitous change in skin odour, which enhances attraction (see PMID: [36200511](https://pubmed.ncbi.nlm.nih.gov/36200511)).
- Was an analysis of this association done controlling for presence of active trachoma, which is also more likely to be found in smaller / lighter kids?
  - As you suggest, it is important to disentangle the effects of age, body weight, and disease – as younger children are more likely to have active trachoma - to answer whether increased fly contact is due to the trachoma or something intrinsically more attractive about smaller children.
  - Therefore, we did control for presence of active trachoma, and the result is presented in table 2 in the “maximally adjusted” column. The association between contacts and body weight was adjusted for age, ocular discharge, and presence of active trachoma. Even after adjusting for these factors the observed association between body weight and fly contacts persisted*.*
  - However, as stated in the discussion, we are measuring different but inter-related variables, and it is difficult to adequately control and measure only a single effect.
- 435: “The youngest children experienced the most fly-eye contact, but among infected children contact levels remained at this higher level for longer (until approximately nine years) than in uninfected children.” This sounds like the description of a nine-year-long longitudinal study, which I don’t think is what is intended.
  - Thank you, we have clarified this (L470)

**Reviewer #2: Dear Editorial staff / Authors,**

The manuscript seems well organized, but kindly you can find some points needs to be revised by the respectable authors, as below:

- The type of study should be mentioned in the “methods” part.
  - There is not one single study design for the results and analysis in this paper. There was a cross-sectional survey performed to collect environmental and demographic characteristics of children, and a laboratory study on Ct carriage in flies. We feel that each element has been described clearly in the methods without the need for a single term to cover it. But we have amended the title to describe this a little more clearly, describing them as “Field- and laboratory-based studies…”
- The manuscript needs to be improved for English language.
  - Thank you. Reviewer 1 pointed out some errors in the language which we have resolved, and we have carefully proofread the document to check for any other issues.
- In abstract it has been written “Fly-eye contact was positively associated with the presence of trachoma disease, lower body weight and increased body temperature.” But the body weight loss and fever have not been defined in this survey.
  - The effect of body weight was measured and analysed, please see L152/L276 (methods).
  - We did not seek to determine a role for fever (>37.5°C), rather, we measured the body temperature of all participants and investigated for associations with fly-eye contact and the subtle differences in body temperature between individuals.
- As mentioned in line 10: “the role of *Chlamydia trachomatis* as vectors has been convincingly demonstrated via randomised controlled trials in the Gambia”, why this study has been performed on a well-known fact?
  - While the role of *M. sorbens* as a vector of Ct in Gambia is known, our contribution adds three important aspects to the literature:
    1. Although the role of *M. sorbens* as vectors was widely accepted through the Gambian studies, that does not mean that in other geographical regions they have the same importance. With this type of (most likely) mechanically transmitted disease, environmental factors and those intrinsic to the fly biology can greatly affect the contribution of flies to transmission. Here, we specifically investigate the role of *M. sorbens* in transmission in Oromia, Ethiopia.
    2. In this paper we also delve into the mechanisms of *how* Ct is transmitted by *M. Sorbens*
    3. We also look at risk factors associated with increased exposure/contact from *M. sorbens*.
- In line 37 “The authors found evidence of on Ct on flies and…” the proposition “on” has been repeated twice.
  - Thank you, amended (L59)
- Considering the Oromia region of Ethiopia as the place of research performing, and contribution of some respected authors outside Ethiopia, it seems better that the role of each author be specified in the manuscript.
  - The role of each of the authors is specified in the “Contributors” section at the end of the manuscript.

**Reviewer 3**

Major concerns:

- The authors failed to deduce the main points from the data that communicate to the scientific community and the title to my understanding requires to capture that. Change the title to suit the obtained data.
  - We agree that the title was not specific enough to communicate the findings of our paper and so we have amended this to “Field- and laboratory-based studies on correlates of *Chlamydia trachomatis* transmission by *Musca sorbens*: determinants of fly-eye contact and investigations into fly carriage of elementary bodies”
- Was there a negative control region for the flies study design?
  - This was an observational study and as such there is no control region (As there was no intervention performed). The entomological aspects of the field study (1) described flies caught at eyes across the whole study, (2) looked at the spatial distribution of Ct positive flies in the study area, (3) measured fly-eye contact and related parameters and (4) roughly tested for where Ct was found on field-caught flies. This work was part of a larger survey, examining trachoma transmission routes more broadly, and involved testing for Ct (at numerous ocular and non-ocular sites including flies) in 247 households. All entomological studies examined infection and/or disease (in the human host or on the fly) and so for both fly-eye contact and Ct carriage by flies we compare (rates) with individuals or households with no Ct (But we wouldn’t characterize those who were Ct-negative as “controls”).
- With regards to the study design of the lab experimental, the data lacks surety of whether the acquired bacterium could be transmissible for no evidence to a suitable animal model, and the authors failed to acknowledge this or cite it as a limitation.
  - We agree that we are showing presence of the bacteria, we are not demonstrating transmissibility. We have acknowledged this in the final paragraph of the paper (“It is also critical to explore onwards transmission routes from the fly: frequent regurgitation and defecation by filth flies allows the deposition of ingested microbes including pathogens.”), and to address your comment we have expanded this paragraph to more clearly set out the limitation (L532)
  - In these studies we did not set out to demonstrate transmissibility, this would not be possible from an ethical standpoint using the natural parasite/vector/host system – we cannot seek to infect a person with ocular Ct. Other studies may investigate this using an animal model however we argue that an animal model concerns different species and, while they may be informative, they do not investigate the actual parasite/vector/host dynamics for this system. Further, transmission experiments are difficult and costly and were outside the scope of this study. We hope that by amending the title, and editing the final paragraph, we remove any confusion that we are presenting controlled transmission experiments.
- Further, if proximity of infection hosts was key to transmission rates, this was not proven or attempted or just ignored in the experimental design.
  - The transmission of any aetiological agent from one host to another, and especially a vector borne disease, can be influenced by many factors relating to the organisms involved and to external factors. Our field studies demonstrated a possibility that the presence of ocular infection of a human host positively influences that number of fly-eye contacts made on that individual. This may have an impact on disease transmission, but there are many other factors that influence onwards disease transmission pertaining to the host, vector, and environment. In this paper we suggest that this phenomenon may influence transmission. In a simple experimental investigation of how the bacterium is carried on the body of the fly it would not be correct to attempt to mimic or control other parameters of transmission without more fully understanding all the factors involved and their relative influence (e.g. host attractiveness, climate on the bacterium longevity, etc.). Such an investigation may be more appropriately conducted through mathematical modelling. In our experimental studies we only wanted to localise the bacterium on or inside the fly, and so we focussed on studying that alone.
- Additionally, the proposed tests in lines 493 – 497 are doable to strengthen manuscript, and therefore recommended.
  - Thank you, we agree that these studies should certainly be carried out and indeed have written funding applications to this end. This is not a trivial amount of work, both in terms of time and financial resources. It would have been fantastic to have included the results of these experiments, but they were not within the scope of the work and we did not have access to the resources that were required.

Minor comments

*Abstract*

- Line 8 ….. are thought to be vectors of
  - Amended
- Line 14 delete ‘flies’ in the brackets
  - Amended

*Introduction*

- Line 71: What is the meaning of ‘E’ interventions, not defined
  - ‘E’ interventions are ‘Environmental’ interventions, the ‘E’ component of the ‘SAFE’ strategy to control trachoma. This has been clarified in the text (L93)

*Methods*

- Study location and design: How many times were the surveys conducted and does this impact how the data was analyzed? What kind of sampling strategy was adopted and the rationale considered? How close or far from each other were the households chosen, and why? These questions were not clearly answered.
  - As indicated (L141) one survey was conducted, running continuously from April to June 2018. Full details regarding this survey is given in a previously published paper (Last, 2020). The survey was in a single location, and there was no sampling of households – all households in the area were included if at least one child aged 1-9 years was resident. This has been added to the study location and design section. The study design section has been slightly expanded however to give more detail (L142)
- Line 136: How were the flies caught killed? Simply say acetone killing
  - Yes, flies were killed using acetone vapours and this has been clarified in the text (L162)
- Line 156: Clearly define the meant specific ‘testing’
  - The headings have been clarified as follows:
    - (L168) Fly identification and testing for Ct DNA (whole flies)
    - (L179) Fly testing for Ct DNA (body parts)
- The authors need to clearly state the number of independent replicates conducted for the fly experiments. Why was the laboratory experiments conducted with very few flies? Does this not limit the power of the
  - The field studies were a survey, we sought to catch and test as many flies per child in the survey as possible. The study was restricted by resources to catching up to 10 flies per child. This is due to the number of children visited/studied per day and the need for an unused, sterilised fly net to catch each fly in. Five flies (per child) were tested whole for Ct DNA according to the financial resources we could allocate towards the fly testing. For the fly body part experiment, all remaining flies (i.e. if more than five flies had been caught per child, the remaining were tested) in the ocular positive households were used, therefore we included all the flies that we had caught that were at a higher likelihood of harbouring Ct.
  - In the laboratory experiments, the first two experiments used three replicates (flies) per time point. This balanced sufficient replication with time and resource limitations. The final experiment (experiment C) used six flies per time point in an attempt to strengthen our findings. We have added a line to note this limitation of our study (L493)
- Fly samples for Ct testing: The authors wrote that ‘knocked-down flies were first washed and transferred into bleach….’ This experiment does not mimic the natural transmission phenomenon. For if the external bacterium on the flies’ surface was washed off, then not detectable for mechanical transmission to be noticed experimentally, or not independently assayed (then contradicts the results lines 377 – 382). Why bleach the samples from the lab and fail to do the same for those from the field, yet a comparison is expected to be made? Any valid reasons?.
  - The laboratory studies are not attempting to mimic natural transmission phenomenon beyond the point of fly feeding and contamination with Ct. Following this, we wanted to localise Ct on or in the fly at two time points, 30 minutes and one hour. With our methodology it was not possible to more precisely locate Ct on the fly, this would require other techniques for example immunofluorescence or sterile dissections. Our described experiments were a relatively ‘quick and dirty’ (yet necessary first step) look at whether Ct is found primarily on the fly’s body, or inside in the head or body (thorax and abdomen). After feeding the fly, we made a ‘wash’ of the fly body surface that we then tested and quantified Ct from. To then test the same fly internally (for head and body Ct), it is essential to bleach the outside surface of the fly to remove any further external contamination. Without the bleaching step we would not be able to localise any Ct detected to the inside of the fly.
  - In the field experiments (fly body part testing) there was no test of an external ‘wash’ relative to internal testing, so there was no need to perform a bleach step. The field experiments were conducted first and were intended, again, to give a ‘quick and dirty’ idea of where on the fly the Ct could be found. For ease, the flies were simply divided into head, ‘body’ and legs.
  - We intend these experiments to be taken together as a set of simple experiments designed to start to tease apart how the fly may be carrying Ct in the field setting.

*Statistical analysis*

- Authors failed to inform how all the fly catches irrespective of infection status were analyzed, based on locations for fly density before stratifying for infection positivity, which is stated in lines 268 – 276. **I recommend re-analysis.**
  - In this paragraph we do not do any analysis as such. We simply report the proportion of flies that were *M. sorbens*, and the sex of the flies. In the subsequent paragraph we mention how many of the flies tested positive, which should not be interpreted as the prevalence of Ct among flies in the area. We emphasise rather the contrast in fly Ct positivity rates between households with ocular positive individuals and those without (L35, 418, 439).
  - We hope that this in part responds to your query, however, we are not entirely clear which analyses you felt were omitted, nor which re-analysis you are proposing
- What was the importance of collecting video data of fly-contacts yet the flies could be counted manually, and or was not presented.
  - Thank you for bringing this to our attention, as it should have been explained. We initially intended the videos to be retrospectively analysed for the duration of fly visits – to determine the total amount of time flies spent at the eye (summed over all visiting flies), as has been done in previous fly-eye studies in The Gambia. However, we found that the fly density and number of visits in this region were so high that this was simply not possible (taking at least 30 minutes, often more, per 10-minute video for analysis). We then used the videos to investigate an alternative means of counting fly-eye contact (not published) aiming to improve on the currently accepted, manual, system and so we continued to video all children to collect this data. We also considered it a robust means of saving fly-eye data in case we needed to return to any measurements.
    - We have summarised this in the statistical methods, L288.

*Results*

- As earlier on stated, the data is sufficiently analyzed, well presented, and described.
- Line 306 …… higher on individuals
  - Amended, L339
- Line 352 - 367 A total of 107 (and not 106) flies (refer to line 281 that stated 24/107). Make the necessary corrections
  - These are two separate data points, with a (confusingly!) similar total number of flies. The first data point (107) refers to the number of flies in ocular positive households that were tested whole for Ct (five whole flies were tested per individual). The second data point (106) refers to the (remaining, of all those caught) flies in ocular positive households that were cut into body parts before testing. The text is therefore correct.

1. Nilsson, M.; De Maeyer, H.; Allen, M. Evaluation of Different Cleaning Strategies for Removal of Contaminating DNA Molecules. Genes 2022, 13, 162. <https://doi.org/10.3390/genes13010162>
2. Last A, Versteeg B, Abdurahman OS, Robinson A, Dumessa G, Aga MA, et al. Detecting extra-ocular Chlamydia trachomatis in a trachoma-endemic community in Ethiopia: Identifying potential routes of transmission. PLoS Negl Trop Dis. 2020;14(3):18–32.
