# Supplementary material for: Field- and laboratory-based studies on correlates of Chlamydia trachomatis transmission by Musca sorbens: Determinants of fly-eye contact and investigations into fly carriage of elementary bodies
Source: PLoS Negl Trop Dis. 2024 Jul 2;18(7):e0012280. doi: 10.1371/journal.pntd.0012280 (PMC11249242; doi:10.1371/journal.pntd.0012280)
Supplement: Attachment — Submitted filename: Response_to_reviews_plosntd.docx [file pntd.0012280.s005.docx]

# Revision 2

Reviewer's Responses to Questions

Reviewer #1: (No Response)

Reviewer #2: Revised manuscript seems convincing and well done.

Reviewer #3: (No Response)

--------------------

<b>Editorial and Data Presentation Modifications?</b></br><br/>

Use this section for editorial suggestions as well as relatively minor modifications of existing data that would enhance clarity. If the only modifications needed are minor and/or editorial, you may wish to recommend “Minor Revision” or “Accept”.

Reviewer #1: Line 34-35: suggest change “lower body weight and increased body temperature” to “lower human body weight and increased human body temperature” to make it clear that it’s not these parameters in the flies that are being referred to.

Amended

Line 48 : “leading cause of infectious blindness worldwide.” Please change to “leading infectious cause of blindness worldwide.” The blindness itself is not infectious.

Amended

Line 58/59: two “found”s in this sentence

Amended

Line 59-61: “The findings suggest that controlling fly populations or preventing fly-eye contact in populations with a high prevalence of trachoma may help control the disease.” I think this is making too much of the data. They’re useful data, but the associations found don’t mean that an apparently relevant intervention will alter prevalence.

Thank you for bringing this to our attention, we have edited to reflect that fly control may be meaningful to trachoma control if further studies provide more conclusive evidence that flies are vectors of trachoma. (L61-64)

Line 88-89: the material in parentheses “(one component of the environmental [E] interventions used to control 89 trachoma)”. This is both awkward from a flow perspective and internally grammatically incorrect: E is environmental improvement, and “environmental interventions” does not really make sense. Suggest delete it.

Thank you, you’re correct that this sentence did not flow well, we have edited as you suggest. (L91-93)

Line 90: I don’t think it’s correct to say that E interventions are rarely implemented. You could say that specific fly control interventions are rarely implemented, or that E interventions are generally the province of actors outside the trachoma elimination programme, or something along those lines, if you want to. There are many organizations, both governmental and non-governmental, that are doing a lot of work to improve access to WASH in rural communities where trachoma is endemic.

Thank you for your thoughts on this, we have amended to specify fly control only. (L92)

Lines 91 and 100 both refer to “gaps in our knowledge”. Can the authors use a different phrasing for one of these instances?

Thank you for bringing this to our attention, amended.

Line 174: suggest indicate why the 13th household was not included; otherwise the reader is left wondering if this was a resource or administrative decision or if the household just refused to be involved. I don’t think this alters interpretation. It just seems an omission to not provide the rationale.

Again, thank you for highlighting this to us, it was due to staff shortages at the time and this has now been detailed in the text. (L177)

Line 192-193: “to detect possible inhibition and provide the RPP30 gene internal control”. I think I don’t fully understand this. Do the authors mean to say, “to detect possible inhibition of PCR *using* the RPP30 gene as in internal control”?

We have modified the sentence to improve clarity (“Eluates were spiked with human DNA obtained from a HEp2 cervical cell line prior to testing, to detect possible inhibition and to provide an internal control (human *RPP30* gene).” (L197)

By spiking the samples with human cells and checking for amplification of a human gene in the PCR reaction, we both test for sources of PCR inhibition (that would have stopped the reaction from working) and generally provide an internal control (all human-spiked samples should have a clear strong signal for *RPP30*)

Line 269: please change the en-dashes (after each use of “inflammation”) to em-dashes, as called for in the annex to the report on the 4th Global Scientific Meeting on Trachoma

Thanks for this correction, amended.

Line 416: suggest change to “person-level” variables

Thanks for this correction, amended.

Line 438: I think “bacterium” should be “bacterial” here

Thanks for this correction, amended.

Line 438 and 439: I am not sure what the authors mean by “competition” in this context

Thanks again, we have made this clearer by stating interspecific competition from the flies’ microbiome (L454-457).

Line 462-464: “but among infected children contact levels were observed at this higher level into older age groups (until approximately nine years) than in uninfected children” – this does not make sense to me. “Than” indicates a comparison. What is the comparison that is being made?

Yes, this sentence was not very clear. We have amended to the following:

Overall, the youngest children experienced the most fly-eye contact. However, while in uninfected children fly-eye contact rates dropped steadily with age, in infected children fly-eye contact rates remained at the higher rates until approximately nine years of age. These data give some indication that in the age range of four to nine years, Ct infection may contribute to increased fly-eye contact rates. (L477-483)

Reviewer #2: (No Response)

Reviewer #3: (No Response)

--------------------

<b>Summary and General Comments</b></br></br>

Use this section to provide overall comments, discuss strengths/weaknesses of the study, novelty, significance, general execution and scholarship. You may also include additional comments for the author, including concerns about dual publication, research ethics, or publication ethics. If requesting major revision, please articulate the new experiments that are needed.

Reviewer #1: This is a nice set of studies that are generally well described in the paper and which contribute significant data. My biggest comment is that - partly due to the number of different kinds of experiment included in an attempt to generate an overall thesis on Ct transmission – I personally found it hard to piece together the evidence for internal carriage of Ct. The abstract says that “Testing for Ct on field-caught M. sorbens indicated a possibly greater role for internal carriage than that on exterior surfaces.” but as I read the body of the paper my impression had been that the evidence on internal carriage actually came from studies on lab-reared flies, or perhaps comparisons of data on lab-reared and field-caught flies. Some more signposting about the significance of particular experiments and the data that came from them would be helpful in this regard.

For example: I think in lines 395-401 the authors are describing analyses on dissected parts of lab-reared flies that (a) had been fed solutions containing Ct, then (b) washed so that any external Ct would be removed and (c) homogenized so that internal Ct DNA would be available for PCR. The results then tell me that Ct DNA was present inside the flies. Is that interpretation correct? I’m sure my uncertainty here is related to unfamiliarity with some of the methods, but to help readers like me it might be helpful just to be a bit more explicit in the results and discussion sections.

Thanks for suggesting that we review this aspect of the paper, which we agree was not sufficiently clear. In response to your comment, we have made extensive amendments to the methods (L179-180; 229-238), results (L405-409) and discussion (L487-527) to clarify our studies and provide more explanation and context. Your interpretation of the lab-reared flies work was correct, but we hope we have now better described the studies. The line you quote in the abstract “Testing for Ct on field-caught M. sorbens indicated a possibly greater role for internal carriage than that on exterior surfaces.” was slightly misleading (overly speculative), as such we have removed it and replaced it with an appropriate statement describing our data (L38-40).

Reviewer #2: (No Response)

Reviewer #3: (No Response)

--------------------

PLOS authors have the option to publish the peer review history of their article (<a href="https://journals.plos.org/plosntds/s/editorial-and-peer-review-process#loc-peer-review-history" target="_blank">what does this mean?</a>). If published, this will include your full peer review and any attached files.<br><br>

If you choose “no”, your identity will remain anonymous but your review may still be made public.<br><br>

<b>Do you want your identity to be public for this peer review?</b> For information about this choice, including consent withdrawal, please see our <a href="https://www.plos.org/privacy-policy" target="_blank">Privacy Policy</a>.

Reviewer #1: No

Reviewer #2: Yes: Hamidreza Hasani

Reviewer #3: No
